# A Novel Nonlinear Piezoelectric Energy Harvesting System Based on Linear-Element Coupling: Design, Modeling and Dynamic Analysis

**DOI:** 10.3390/s18051492

**Published:** 2018-05-09

**Authors:** Shengxi Zhou, Bo Yan, Daniel J. Inman

**Affiliations:** 1 School of Aeronautics, Northwestern Polytechnical University, Xi’an 710072, China; 2 Faculty of Mechanical Engineering & Automation, Zhejiang Sci-Tech University, Hangzhou 310018, China; 3 Department of Aerospace Engineering, University of Michigan, Ann Arbor, MI 48109-2140, USA; daninman@umich.edu

**Keywords:** linear elements, coupled system, modeling, energy harvesting, nonlinear dynamics

## Abstract

This paper presents a novel nonlinear piezoelectric energy harvesting system which consists of linear piezoelectric energy harvesters connected by linear springs. In principle, the presented nonlinear system can improve broadband energy harvesting efficiency where magnets are forbidden. The linear spring inevitably produces the nonlinear spring force on the connected harvesters, because of the geometrical relationship and the time-varying relative displacement between two adjacent harvesters. Therefore, the presented nonlinear system has strong nonlinear characteristics. A theoretical model of the presented nonlinear system is deduced, based on Euler-Bernoulli beam theory, Kirchhoff’s law, piezoelectric theory and the relevant geometrical relationship. The energy harvesting enhancement of the presented nonlinear system (when *n* = 2, 3) is numerically verified by comparing with its linear counterparts. In the case study, the output power area of the presented nonlinear system with two and three energy harvesters is 268.8% and 339.8% of their linear counterparts, respectively. In addition, the nonlinear dynamic response characteristics are analyzed via bifurcation diagrams, Poincare maps of the phase trajectory, and the spectrum of the output voltage.

## 1. Introduction

In order to solve the challenging issue of the energy supply for wireless sensors, small portable devices and MEMS, piezoelectric vibration energy harvesting based on the piezoelectric effect has been receiving more and more attention over the past two decades [1,2,3,4,5,6,7,8,9,10]. Meanwhile, piezoelectric vibration energy harvesting will positively promote the development of the structural health monitoring and the precision actuation [11,12,13,14,15,16,17,18]. Up to now, many different kinds of linear resonance based piezoelectric vibration energy harvesters have been designed, modeled, simulated and experimentally tested to investigate their energy harvesting performance [19,20,21,22,23,24]. However, these resonance based linear piezoelectric vibration energy harvesters are very sensitive to the external excitation frequency, which leads to the reduced capacity of vibration energy harvesting when they are subjected to a wide range of excitation frequencies. This issue has been inspiring many researchers to focus on widening the operating bandwidth of the vibration energy harvesters and enhancing their energy harvesting performance based on the active and adaptive frequency-tuning schemes [25,26,27,28,29,30,31,32,33,34].

Currently, intensive investigation is focusing on magnet-based nonlinear energy harvesting in the purpose of widening the operating frequency range and enhancing the energy harvesting performance. It was demonstrated that magnet-based nonlinear monostable energy harvesters have a wider effective bandwidth and a higher energy harvesting efficiency than their linear counterparts [35,36,37,38,39]. What’s more, the advantages of high-energy interwell oscillations and the broadband operating frequency range of the bistable configurations have been employed to harvest energy from broadband base excitations [40,41,42,43,44,45,46]. Recently, tristable energy harvesters with suitable design parameters were introduced and they experimentally exhibited a better energy harvesting performance compared to bistable energy harvesters under a very low level excitation [47,48,49,50,51,52,53]. These magnet-based nonlinear energy harvesters have been verified that they have an excellent energy harvesting performance and the broadband characteristics. However, in some special application areas (where magnets have the undesirable influence to the host objects or ambient environment), magnets are forbidden. Therefore, investigation of the non-magnet based vibration energy harvesting enhancement technique is necessary and meaningful.

For the non-magnet based vibration energy harvesting enhancement technique, Leland and Wright [54] presented a resonance-changeable piezoelectric vibration energy harvester with an adjustable axial preload. This harvester provides a wider operating frequency range than that of traditional linear energy harvesters. Shahruz et al. [55] presented a broadband piezoelectric vibration energy harvesting system by gathering several linear energy harvesters with different resonant frequencies together. Kim et al. [56] and Wu et al. [57] separately proposed a two degree of freedom (2-DOF) energy harvesting system, and their results showed that such systems have two peak displacement amplitudes at two different resonant frequencies causing a wider operating frequency range than that of the linear 1-DOF energy harvester. Kuch and Karami [58] provided a theoretical model of a nonlinear hybrid rotary-translational energy harvester and explored the application for powering heart pacemakers. Liu et al. [59] designed a bistable piezoelectric energy harvester based on a buckled spring-mass system, and their results show that a maximum power of 16 mW could be obtained for a 0.3 g chirp excitation. Chen et al. [60,61] utilized the internal resonance mechanism to enhance vibration-based energy harvesting, and they made a theoretical analysis via nonlinear methods. Xu and Tang [62] developed a cantilever-pendulum energy harvesting system, which could harvest vibration energy of excitations from three directions in simulation. Li et al. [63] numerically and experimentally verified the broadband characteristics of a compressive-mode energy harvester. Wei and Jing [64] proposed a nonlinear energy harvesting system via a lever system combined with an X-shape supporting structure, and the numerical results show that this system provides a great flexibility and/or a unique tool for tuning and improving energy harvesting efficiency via matching excitation frequencies and covering a wider frequency range.

Previous research theoretically and experimentally verified on the enhanced performance of the non-magnet based vibration energy harvesting technique [54,55,56,57,58,59,60,61,62,63,64]. More importantly, non-magnet based vibration energy harvesting has an irreplaceable application potential in some special fields where magnets are forbidden. Meanwhile, more research and investigations about the non-magnet based vibration energy harvesting technique are need to promote the development and application of vibration energy harvesting. Therefore, it is meaningful to present new concepts or structures based non-magnet based nonlinear energy harvesting technique to enhance vibration energy harvesting performance.

This paper presented a novel nonlinear piezoelectric energy harvesting system (NPEHS) based on linear-element coupling, and it contains linear piezoelectric energy harvesters connected by linear springs. In Section 2, a theoretical model of the presented NPEHS is derived based on Euler-Bernoulli beam theory, Kirchhoff’s law, piezoelectric theory, and the assumed geometrical relationship. In Section 3, case study is provided. In Section 4, the nonlinear dynamic response characteristics of the presented NPEHS are analyzed via bifurcation diagrams, Poincare maps of the phase trajectory, and the spectrum of the output voltage. Finally, key conclusions are presented.

## 2. Concept and Modeling

**Figure 1:**
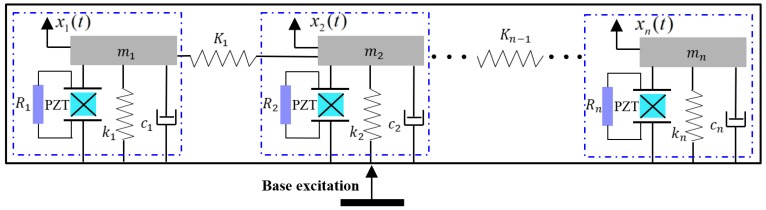
Schematic diagram of the NPEHS.

**Figure 2:**
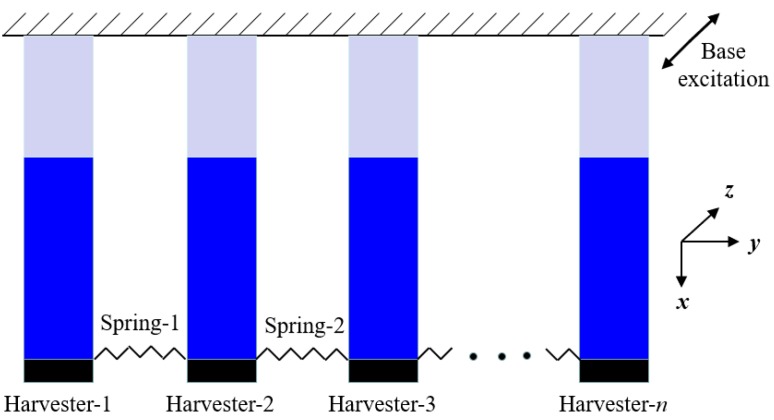
Structural diagram of the NPEHS.

The idea of the presented NPEHS is based on the linear element coupled system, as its schematic diagram shown in Figure 1. The equivalent model of each linear energy harvester is surrounded by the closed blue dotted line, which was explored in Ref. [3]. Although each independent energy harvester has linear characteristics, based on coupled dynamic behaviors [65,66,67], the whole system will exhibit nonlinear characteristics when two adjacent linear energy harvesters are connected by linear springs (*K*_1_, … *K_n_*_−1_ stand for their stiffness). Figure 2 shows the structure diagram of the presented NPEHS. In principle, the NPEHS contains *n* linear piezoelectric energy harvesters with different resonant frequencies, and they are connected by *n* − 1 linear springs. The base excitation is imposed in the *z* direction, which is the same direction of the bending vibration of each energy harvester of the NPEHS. All the linear springs are connected in the *y* direction (width direction of each energy harvester). The NPEHS will exhibit nonlinear dynamic response characteristics when it is subjected to an excitation, because the nonlinear spring force is inevitably produced by the linear springs (which are disproportionately extended by the different vibration displacements from two connected adjacent energy harvesters in the system). The bending stiffness for a cantilever beam with rectangular cross section is $EI=\frac{bE_{}h_{}^{3}}{12}$. Therefore, the ratio of the bending stiffness for the lateral motion (*y* direction) *EI_y_* and the bending stiffness for the transverse motion (*z* direction) *EI_z_* is $\frac{EI_{y}}{EI_{z}}={\left(\frac{b}{h}\right)}^{2}$. In this paper, the width *b* of the substrate is 65 times more than of the thickness *h*, which will be given in Table 1 in Section 3. Therefore, *EI_y_* is more than 4000 times of *EI_z_*. In this case, the lateral motion is negligible. It is truly that the harvester will become softer in the *y* direction as the stiffness of the connected spring increases. However, the stiffness of the connected spring is much smaller than the lateral equivalent stiffness in this study.

In this paper, a theoretical model of the NPEHS is deduced based on Euler-Bernoulli beam theory, piezoelectric theory, Kirchhoff’s law, and the relevant geometric relationship. In the theoretical model, we assume that all the linear springs are elastic and own the constant stiffness.

**Figure 3:**
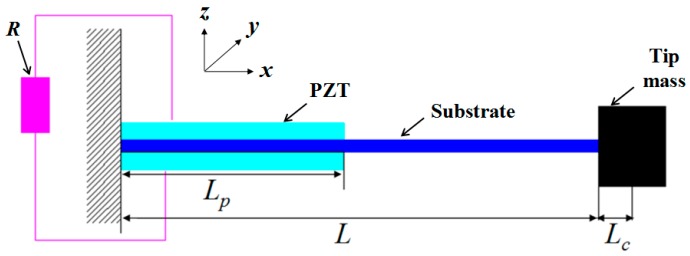
Linear piezoelectric energy harvester with the non-uniform cross-section in the NPEHS.

In order to obtain the theoretical model of the presented NPEHS under a base excitation, the electromechanical governing equations of each linear energy harvester should be firstly built based on Euler-Bernoulli beam theory, proportional damping, piezoelectric theory, and Kirchhoff’s law. In this study, each linear piezoelectric energy harvester in the proposed system has the non-uniform cross-section as its schematic shown in Figure 3. The length of the substrate and the piezoelectric layers of the drawn harvester is *L* and *L_p_* (in the *x* direction), respectively. The thickness of the former and the latter is *h_s_* and *h_p_* (in the *z* direction), respectively. *L_c_* is the half length of the cuboid tip mass block. *R* is the external resistance load. *b* is the width (in the *y* direction) of both the substrate and the piezoelectric layers.

As one of the mechanisms for energy conversion, piezoelectric laminates bonded to cantilever beams have been widely studied. The piezoelectric constitutive equations are used to describe their electromechanical behavior, as shown in Appendix A. For a thin cantilever beam with the uniform cross-section, these parameters are given by Erturk and Inman [3]. Based on Euler-Bernoulli beam theory, piezoelectric effect, and Kirchhoff’s law, the electromechanical governing equations of a linear piezoelectric energy harvester with non-uniform cross-section can be written as:

(1) $$\begin{array}{l} EI\frac{\partial^{4}v(x,t)}{\partial x^{4}}+c_{s}\frac{\partial^{5}v(x,t)}{\partial x^{4}\partial t}+c_{m}\frac{\partial v(x,t)}{\partial t}+m\frac{\partial^{2}v(x,t)}{\partial t^{2}}-\vartheta V(t)\left[\frac{d\delta (x)}{dx}-\frac{d\delta (x-L_{p})}{dx}\right]=\ldots \\ -\left[m+M_{tip}\delta (x-L)+M_{tip}L_{c}\frac{d\delta (x-L)}{dx}\right]\frac{\partial^{2}v_{b}(t)}{\partial t^{2}} \end{array}$$

(2) $$C_{p}\frac{dV(t)}{dt}+\frac{V(t)}{R}+\vartheta \int_{0}^{L_{p}}\frac{\partial^{3}v(x,t)}{\partial x^{2}\partial t}dx=0$$

where $v_{b}(t)$ is the base displacement used as the excitation; v(x,t) is the displacement of the energy harvester relative to the base; V(t) is the output voltage across *R*; $c_{m}$ and $c_{s}$ are the external damping coefficient (mass-proportional damping) and the internal damping coefficient of the composite structure (stiffness-proportional damping), respectively. $M_{tip}$ is the tip mass; the equivalent capacitance of the piezoelectric layers for parallel connection in this paper is $C_{p}=2\varepsilon_{33}^{S}bL_{p}/h_{p}$; the electromechanical coupling term is $\vartheta =e_{31}b(h_{s}+h_{p})$ for parallel connection of the piezoelectric layers; *m* and *EI* are the mass per unit length of and the bending stiffness of the energy harvester, respectively. They depend on the location of the piezoelectric layers, as follows:$m_{1}=b\rho_{s}h_{s}+2b\rho_{p}h_{p}$, for $0\leq x\leq L_{p}$; $m_{2}=b\rho_{s}h_{s}$, for $L_{p}<x\leq L$; $EI_{1}=\frac{2}{3}b\left(\frac{E_{s}h_{s}^{3}}{8}+E_{p}\left({\left(h_{p}+\frac{h_{s}}{2}\right)}^{3}-\frac{h_{s}^{3}}{8}\right)\right)$, for $0\leq x\leq L_{p}$; $EI_{2}=\frac{bE_{s}h_{s}^{3}}{12}$, for $L_{p}<x\leq L$. $\rho_{p}$ and $\rho_{s}$ are the density of the piezoelectric layers and the substrate, respectively; $h_{p}$ and $h_{s}$ are the thickness of the piezoelectric layers and the substrate, respectively; $E_{p}$ and $E_{s}$ are the Young’s modulus of the piezoelectric layers and the substrate, respectively.

The relative displacement v(x,t) in the physical coordinates can be written as the combination of the mode shape $\phi_{i}(x)$ and the modal coordinates $r_{i}(t)$, as follows:

(3) $$v(x,t)=\sum_{i=1}^{n}\phi_{i}(x)r_{i}(t)$$

Since the piezoelectric layers are not covering the whole beam, the mode shape of the energy harvester is comprised of two different parts:

(4) $${\left(\phi (x)\right)}_{1}=A_{1}\sin(\beta_{1}x)+B_{1}\cos(\beta_{1}x)+C_{1}\sinh(\beta_{1}x)+D_{1}\cosh(\beta_{1}x),\;\mathrm{for}\;0\leq x\leq L_{p}$$

(5) $${\left(\phi (x)\right)}_{2}=A_{2}\sin(\beta_{2}x)+B_{2}\cos(\beta_{2}x)+C_{2}\sinh(\beta_{2}x)+D_{2}\cosh(\beta_{2}x),\;\mathrm{for}\;L_{p}<x\leq L$$

The eigenvalue equations are given by:

(6) $$EI_{1}{\left(\phi \right)}_{1}^{iv}-m_{1}\omega^{2}{\left(\phi \right)}_{1}=0$$

(7) $$EI_{2}{\left(\phi \right)}_{2}^{iv}-m_{2}\omega^{2}{\left(\phi \right)}_{2}=0$$

where $\omega =\beta_{1}^{2}\sqrt{\frac{EI_{1}}{m_{1}}}=\beta_{2}^{2}\sqrt{\frac{EI_{2}}{m_{2}}}$.

At the clamped end, the displacement and the angle of rotation should be zero. Since the linear piezoelectric energy harvester is considered to meet Euler-Bernoulli beam theory, the condition of the displacement, the angle of rotation, the bending moment and the shear force are continuous at the joint position of two different parts.

Based on boundary conditions shown in Appendix A, orthogonality conditions of the normalized mode shapes can be used to get the final dynamic model:

(8) $$\begin{array}{l} \int_{0}^{L_{p}}{\left(\phi_{i}(x)\right)}_{1}m_{1}{\left(\phi_{j}(x)\right)}_{1}dx+\int_{L_{p}}^{L}{\left(\phi_{i}(x)\right)}_{2}m_{2}{\left(\phi_{j}(x)\right)}_{2}dx+{\left(\phi_{i}(L)\right)}_{2}M_{tip}{\left(\phi_{j}(L)\right)}_{2}+\ldots \\ {\left(\phi_{i}(L)\right)}_{2}^{\prime }(I_{t}+M_{tip}L_{c}^{2}){\left(\phi_{j}(L)\right)}_{2}^{\prime }+{\left(\phi_{i}(L)\right)}_{2}M_{tip}L_{c}{\left(\phi_{j}(L)\right)}_{2}^{\prime }+\ldots \\ {\left(\phi_{i}(L)\right)}_{2}^{\prime }M_{tip}L_{c}{\left(\phi_{j}(L)\right)}_{2}=\delta_{ij} \end{array}$$

(9) $$\int_{0}^{L_{p}}{\left(\phi_{i}(x)\right)}_{1}^{\prime \prime }EI_{1}{\left(\phi_{j}(x)\right)}_{1}^{\prime \prime }dx+\int_{L_{p}}^{L}{\left(\phi_{i}(x)\right)}_{2}^{\prime \prime }EI_{2}{\left(\phi_{j}(x)\right)}_{2}^{\prime \prime }dx=\delta_{ij}\omega^{2}$$

where *i* and *j* present the vibration modes. $\delta_{ij}$ is the Kronecker delta, which is defined as unity when *i* is equal to *j* and zero otherwise.

In principle, the relative displacement v(x,t) in the physical coordinates consists of an unlimited number of the mode shape $\phi_{i}(x)$ and the modal coordinates $r_{i}(t)$ (*i* = 1, 2, 3, …, *n*), as shown in Equation (3). Meanwhile, the first vibration mode of piezoelectric energy harvesters was theoretically and experimentally verified to play an overwhelming role in vibration energy harvesting [1,3,8,68,69]. Therefore, this study only considers the first vibration mode. The electromechanical governing equations is reduced to only include the first vibration mode of the energy harvester. Based on above derivation, the electromechanical governing equations in the first-order modal coordinates are obtained, as follows:

(10) $$\ddot{r}(t)+2\zeta \omega \dot{r}(t)+\omega^{2}r(t)-\theta V(t)=f(t)$$

(11) $$C_{p}\dot{V}(t)+\frac{V(t)}{R}+\theta \dot{r}(t)=0$$

where the modal electromechanical coupling coefficient $\theta =\vartheta ({\left(\phi (L_{p})\right)}_{1}^{\prime }-{\left(\phi (0)\right)}_{1}^{\prime })$. ζ is the equivalent modal damping ratio, which is based on empirical values in experiments and models [3,70,71].

The modal force f(t) is defined as the following equation:

(12) $$f(t)=-\left[\int_{0}^{L_{p}}m_{1}{\left(\phi (x)\right)}_{1}dx+\int_{L_{p}}^{L}m_{2}{\left(\phi (x)\right)}_{2}dx+M_{tip}{\left(\phi (L)\right)}_{2}+M_{tip}L_{c}{\left(\phi (L)\right)}_{2}^{\prime }\right]\frac{\partial^{2}v_{b}(t)}{\partial t^{2}}$$

**Figure 4:**
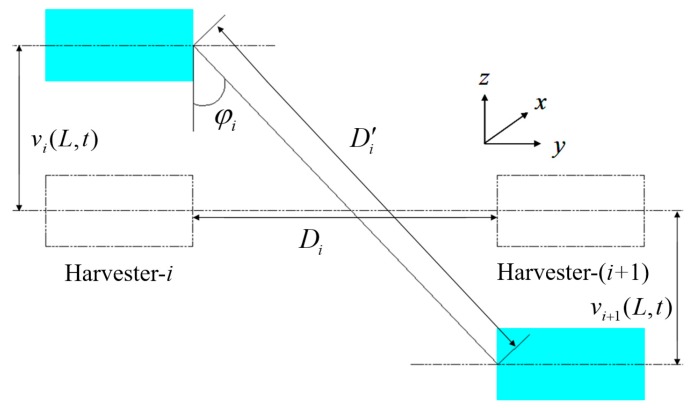
Relative geometrical position of two adjacent harvesters.

By far, the modeling process of the linear piezoelectric energy harvester is completed. In order to get the complete theoretical model of the proposed system, the connected springs should be considered. The detailed geometrical relationship of two adjacent energy harvesters is assumed as the schematic diagram depicted in Figure 4. Since the vibration direction is in the *z* direction, $D_{i}$ is the original length of spring-*i.* $v_{i}(L,t)$ and $v_{i+1}(L,t)$ are the tip displacement of harvester-*i* and harvester-(*i*+1) relative to the base, respectively.

As shown in Figure 4, the transient length of spring-*i* is $D_{i}^{\prime }$, as follows:

(13) $$D_{i}^{\prime }=\sqrt{D_{i}^{2}+{\left(v_{i}(L,t)-v_{i+1}(L,t)\right)}^{2}}$$

The transient included angle $\varphi_{i}$ between spring-*i* and the *z* axis can be written as:

(14) $$\cos(\varphi_{i})=\frac{v_{i}(L,t)-v_{i+1}(L,t)}{D_{i}^{\prime }}$$

Assuming all the springs being elastic and owning constant stiffness, based on Hooke’s law, the effective force generated by spring-*i* upon harvester-*i* is its component force in the *z* direction, as follows:

(15) $$F_{(i+1)i}=K_{i}(D_{1}^{\prime }-D_{i})\cos(\varphi_{i})$$

where $K_{i}$ is the linear stiffness of spring-*i*.

Similarly, the effective spring force between any two adjacent energy harvesters can be calculated. In this study, each energy harvester separately connects with an external load resistance. Therefore, there are *n* mutually independent electrical equations based on Kirchhoff’s law. Finally, the theoretical model of the NPEHS with *n* linear energy harvesters in the first-order modal coordinate system are obtained and described by the following equations:

(16) $$\left\{ \begin{array}{l} \left\{ \begin{array}{l} {\ddot{r}}_{1}(t)+2\zeta_{1}\omega_{1}{\dot{r}}_{1}(t)+\omega_{1}^{2}r_{1}(t)-\theta_{1}V_{1}(t)+{\left(\phi (L)\right)}_{1(2)}F_{21}=f_{1}(t)\\ {\left(C_{p}\right)}_{1}{\dot{V}}_{1}(t)+\frac{V_{1}(t)}{R_{1}}+\theta_{1}{\dot{r}}_{1}(t)=0 \end{array}\right.\\ \left\{ \begin{array}{l} {\ddot{r}}_{2}(t)+2\zeta_{2}\omega_{2}{\dot{r}}_{2}(t)+\omega_{2}^{2}r_{2}(t)-\theta_{2}V_{2}(t)-{\left(\phi (L)\right)}_{2(2)}F_{21}+{\left(\phi (L)\right)}_{2(2)}F_{32}=f_{2}(t)\\ {\left(C_{p}\right)}_{2}{\dot{V}}_{2}(t)+\frac{V_{2}(t)}{R_{2}}+\theta_{2}{\dot{r}}_{2}(t)=0 \end{array}\right.\\ \left\{ \begin{array}{l} \ldots \\ \ldots \end{array}\right.\\ \left\{ \begin{array}{l} {\ddot{r}}_{n}(t)+2\zeta_{n}\omega_{n}{\dot{r}}_{n}(t)+\omega_{n}^{2}r_{n}(t)-\theta_{n}V_{n}(t)-{\left(\phi (L)\right)}_{n(2)}F_{n(n-1)}=f_{n}(t)\\ {\left(C_{p}\right)}_{n}{\dot{V}}_{n}(t)+\frac{V_{n}(t)}{R_{n}}+\theta_{n}{\dot{r}}_{n}(t)=0 \end{array}\right. \end{array}\right.$$

where the subscripts 1, 2, …, *n* stand for the number of energy harvesters in the NPEHS. For example, $F_{i(i-1)}$ is the effective force generated by spring-(*i* − 1) upon harvester-(*i* − 1). Meanwhile, ${\left(\phi (L)\right)}_{i(2)}$ stands for the mode shape of the second part (at the location of *L*) of the *i*-th energy harvester.

## 3. Case Study for Verifying Energy Harvesting Improvement

**Figure 5:**
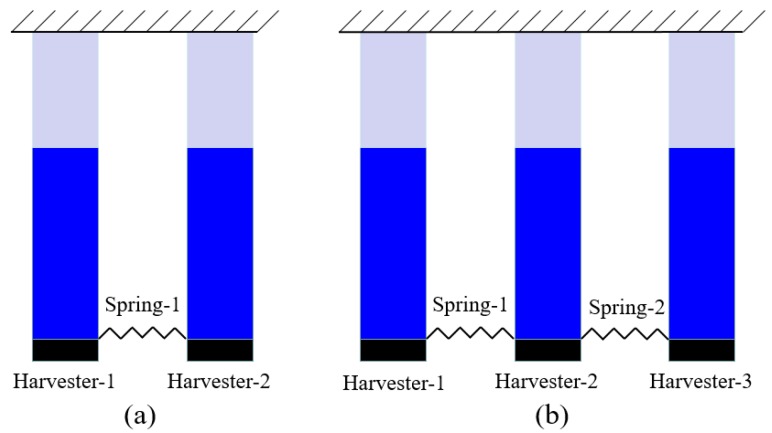
Diagrams of (**a**) the NPEHS-1; (**b**) the NPEHS-2.

In the last section, the theoretical model of the NPEHS is derived. In order to verify its energy harvesting enhancement, the NPEHS-1 (*n* = 2) and the NPEHS-2 (*n* = 3) are investigated below, and their diagrams are shown in Figure 5a,b, respectively. The geometrical parameters of each harvester are shown in Table 1. The material property parameters are depicted in Table 2. In detail, beryllium bronze (UNS C1720) is selected as the substrate, whose density and Young’s modulus are 8250 kg/m^3^ and 125 GPa, respectively. Piezoelectric laminate properties are referred to [45]. The tip mass of each harvester is made of the same material with the substrate, and its size is 15 × 10 × 5 mm^3^. The natural frequency of harvester-1, harvester-2 and harvester-3 is calculated to be 2.59 Hz, 6.18 Hz and 8.86 Hz, respectively. In this section, 0.2 g is selected as the harmonic base excitation level. In addition, linearly increasing frequency excitation simulations with a low rate of frequency change (0.03 Hz/s) are performed.

Under a harmonic base excitation, the output power of each energy harvester can be calculated by using the equation $P={\left(\frac{\sqrt{2}}{2}V_{A}\right)}^{2}/R$ ($V_{A}$ is the output voltage amplitude). By far, there is no generally applicable criterion to determine the energy harvesting capacity. Over a wide range of excitation frequencies, the total area of the output power of an energy harvesting system may stand for its energy harvesting capacity [21]. In addition, the specific value of the energy harvesting improvement of the NPEHS over the linear energy harvesters is very important to estimate its contribution. Therefore, the output power area ratio γ can be used to evaluate the energy harvesting performance of the NPEHS relative to its linear counterparts, as follows:

(17) $$\gamma =P/P_{L}$$

where *P* and $P_{L}$ are the total output power area of the NPEHS and the corresponding linear counterparts, respectively.

### 3.1. The NPEHS-1 with Two Energy Harvesters

Compared with energy harvesters in the system, the connected linear springs are easier to optimize and select. If finding the maximum γ is the optimization objective, the connected linear springs can be optimized by using Genetic Algorithm, Particle Swarm Optimization or other optimization algorithms. A simplest way to optimize the connected linear springs is to calculate the energy harvesting performance over a wide range of parameter values of the springs, and then we can find the best parameters of the springs. In detail, γ of the NPEHS-1 with different springs (initial length and stiffness can be confined in a certain range) can be calculated. However, the other parameters of the NPEHS-1 should be set as constant, when we optimize the springs.

**Figure 6:**
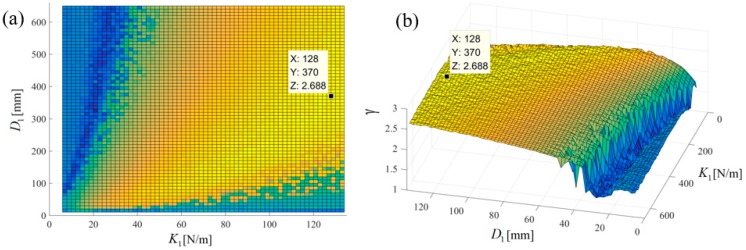
The output power area ratio γ along with different $D_{1}$ and $K_{1}$ of the NPEHS-1: (**a**) Top view; (**b**) oblique view.

**Figure 7:**
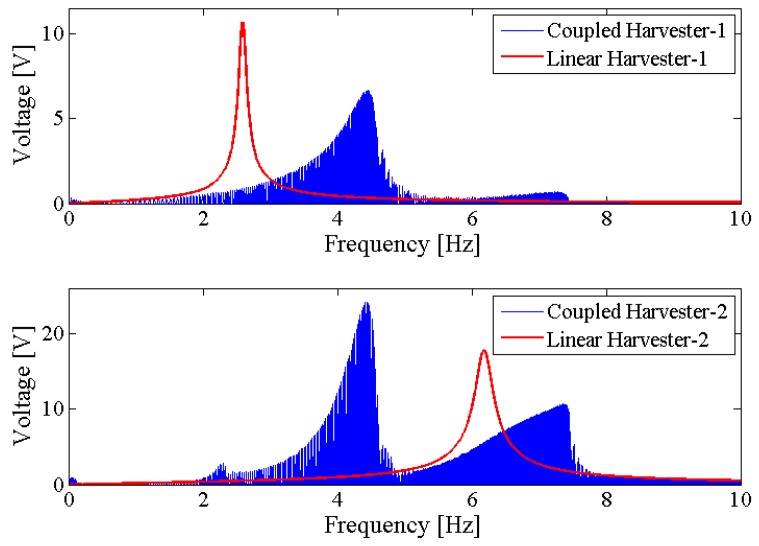
Output voltage of the NPEHS-1.

**Figure 8:**
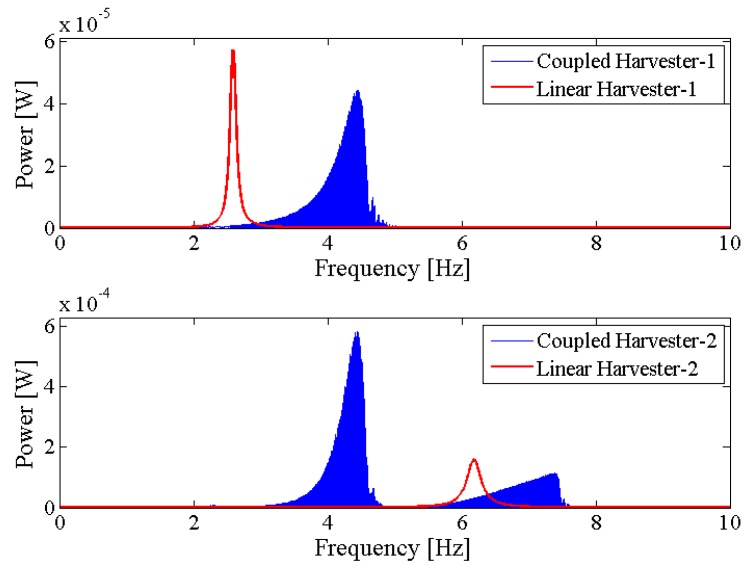
Output power of the NPEHS-1.

Figure 6 shows the output power area ratio γ of the NPEHS-1 along with different $D_{1}$ (ranging from 6 mm to 134 mm) and $K_{1}$ (ranging from 10 N/m to 650 N/m). In detail, Figure 6a,b are the top view and the oblique view of the output power area ratio γ along with different $D_{1}$ and $K_{1}$ of the NPEHS-1. The brighter the area stand for the higher γ. It is found that the optimal initial length $D_{1}$ and the optimal stiffness $K_{1}$ of the connected spring-1 in the NPEHS-1 are 128 mm and 370 N/m, respectively. In this case, γ is 2.688, which means that the output power area of the NPEHS-1 is 268.8% of that of its linear counterparts. The ratio of the physical stiffness of the harvester-*i* and the stiffness of connected spring can be approximate to $\frac{k_{i}}{K_{i}}=\frac{\omega_{i}^{2}}{{\left(\phi (L)\right)}_{i(2)}K_{i}}$, and $\frac{\omega_{1}^{2}}{{\left(\phi (L)\right)}_{1(2)}K_{1}}$ and $\frac{\omega_{2}^{2}}{{\left(\phi (L)\right)}_{2(2)}K_{1}}$ are 0.086 and 0.70, respectively. As the output voltage shown in Figure 7, the NPEHS-1 with optimal spring-1 can efficiently harvest vibration energy over a wider range of excitation frequencies. In addition, the response voltage curve of coupled harvesters in the NPEHS-1 shows obvious nonlinear dynamic response characteristics, which can be identified by comparing with the response voltage curve of linear harvesters in the same plots. The energy harvesting enhancement of the NPEHS-1 can be found in the output power curves, as shown in Figure 8.

**Figure 9:**
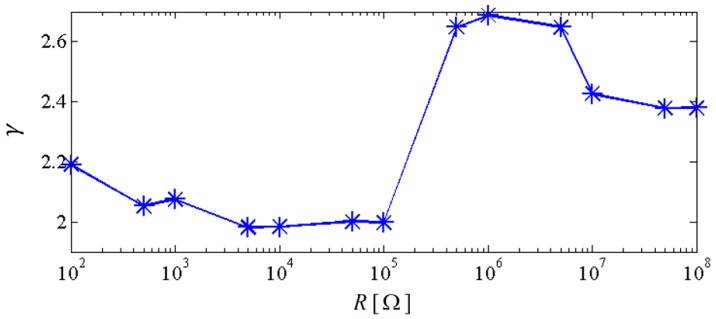
Output power area ratio of the NPEHS-1 versus load resistance.

If the excitation frequency range where the output power is larger than 20 μW is considered to be effective, the effective bandwidth of the coupled harvester-1 is 0.45 Hz which is 321% of that (0.14 Hz) of the linear harvester-1, as shown in the first plot of Figure 8. Meanwhile, the bandwidth of the coupled harvester-2 is 4.86 Hz, while the bandwidth shrinks to be 0.86 Hz in its linear case, as shown in the second plot of Figure 8. This demonstrates that the bandwidth of the NPEHS-1 is wider than its linear counterparts. Figure 9 shows the different output power area ratio γ of the NPEHS-1 subject to different load resistance *R* (ranging from 100 Ω to 10^8^ Ω), and each γ is larger than 1.9. These results further verify the improvement of the energy harvesting performance of the NPEHS-1.

### 3.2. The NPEHS-2 with Three Energy Harvesters

**Figure 10:**
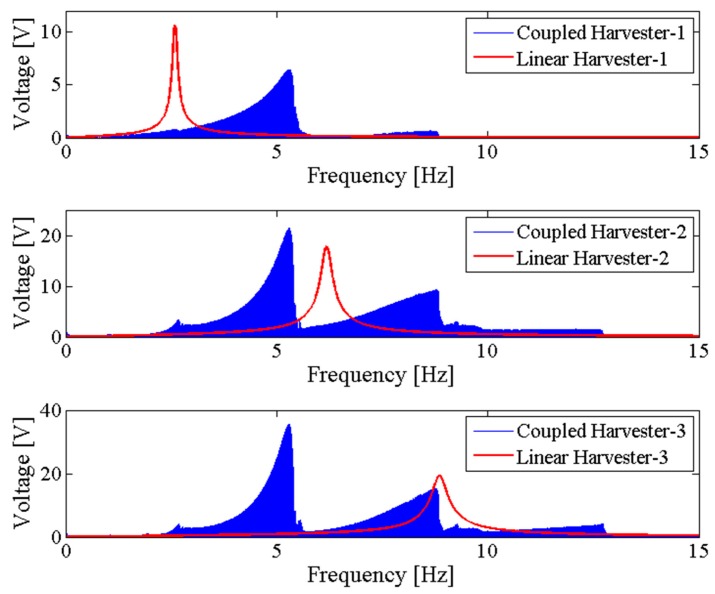
Output voltage of the NPEHS-2.

**Figure 11:**
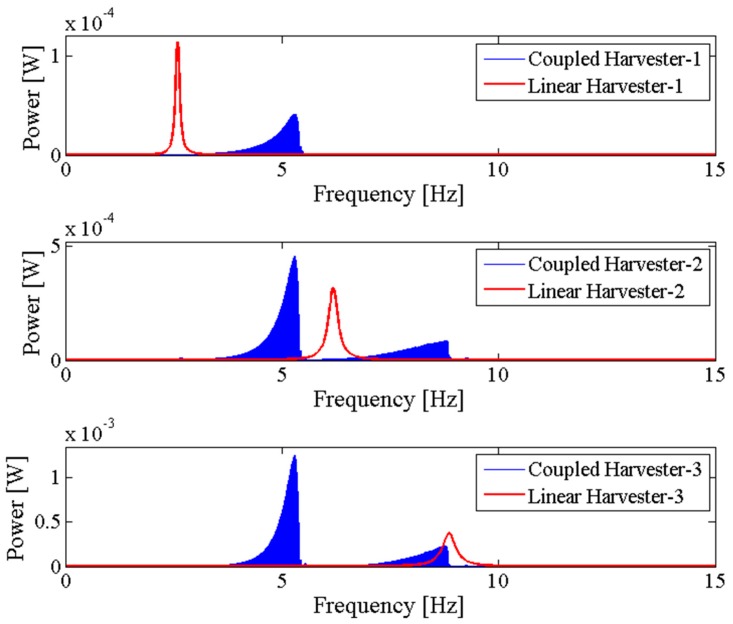
Output power of the NPEHS-2.

**Figure 12:**
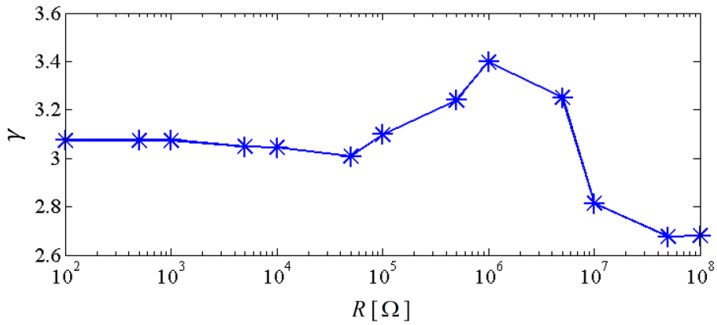
Output power area ratio of the NPEHS-2 versus load resistance.

In order to further examine the energy harvesting enhancement of the proposed system, the NPEHS-2 which consists of harvester-1, harvester-2, harvester-3, spring-1 and spring-2 is studied. Based on calculation, 320 N/m and 78 mm are the optimal stiffness and the optimal initial length of spring-1, respectively. 180 N/m and 8 mm are the optimal stiffness and the optimal initial length of spring-2, respectively. The ratio of the physical stiffness of the harvester-3 and the stiffness of spring-2 is about 3.371. The comparison of the NPEHS-2 and its linear counterparts is shown in Figure 10 and Figure 11. The NPEHS-2 exhibits nonlinear dynamic response characteristics, since the voltage response curves in Figure 10 have obvious frequency-jump phenomena [35,36,37,38,39]. Such nonlinear characteristics can efficiently improve the vibration energy harvesting capacity. In this case, the output power area ratio γ is 3.398, which is larger than that of the NPEHS-1. If the excitation frequency range where the output power is larger than 20 μW is considered to be effective, the effective bandwidth of the coupled harvester-1, the coupled harvester-2, and the coupled harvester-3 of the NPEHS-1 is 0.52 Hz, 3.30 Hz and 3.51 Hz, respectively, as the output power shown in Figure 11. However, the effective bandwidth of the linear harvester-1, the linear harvester-2 and the linear harvester-3 is only 0.14 Hz, 0.86 Hz and 1.53 Hz, respectively. Figure 12 shows γ of the NPEHS-2 subject to different load resistance *R* (ranging from 100 Ω to 10^8^ Ω), and each γ is larger than 2.6. These results demonstrate that the optimized NPEHS with three energy harvesters has a better energy harvesting performance than its linear counterparts.

## 4. Nonlinear Dynamic Analysis

**Figure 13:**
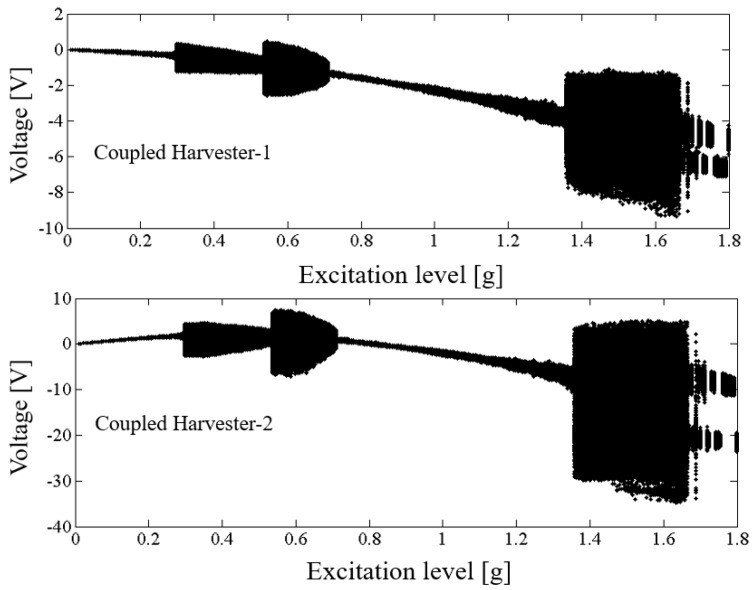
Bifurcation diagram of response voltages versus excitation levels.

**Figure 14:**
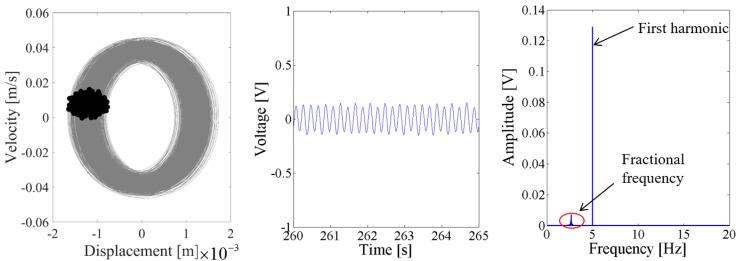
Dynamic responses of the coupled harvester-1 for the excitation level of 0.1 g.

**Figure 15:**
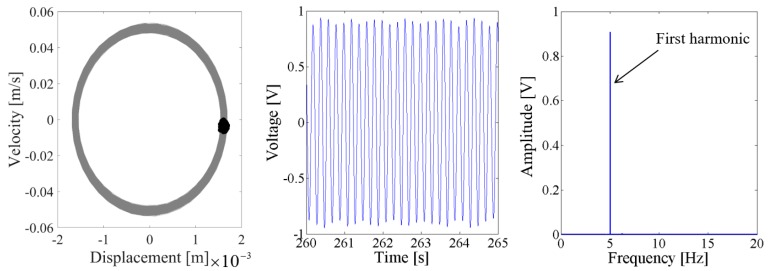
Dynamic responses of the coupled harvester-2 for the excitation level of 0.1 g.

**Figure 16:**
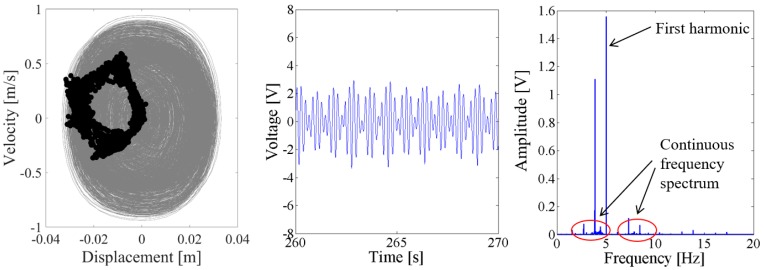
Dynamic responses of the coupled harvester-1 for the excitation level of 0.6 g.

**Figure 17:**
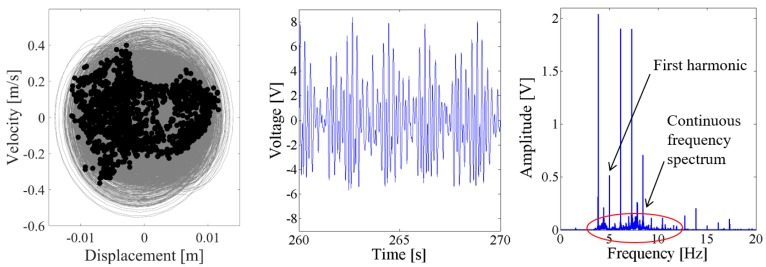
Dynamic responses of the coupled harvester-2 for the excitation level of 0.6 g.

**Figure 18:**
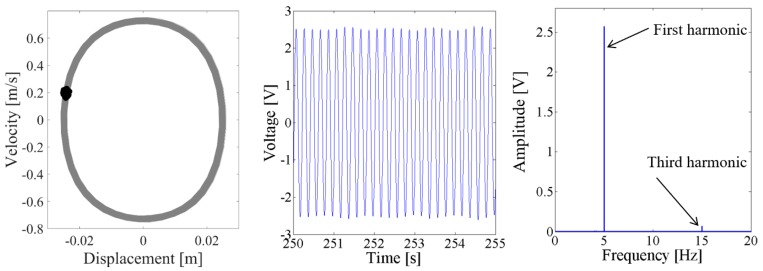
Dynamic responses of the coupled harvester-1 for the excitation level of 0.9 g.

**Figure 19:**
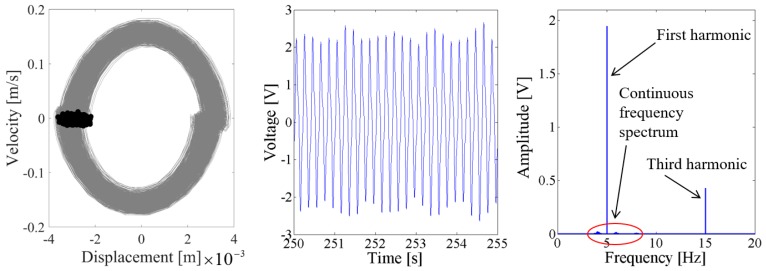
Dynamic responses of the coupled harvester-2 for the excitation level of 0.9 g.

**Figure 20:**
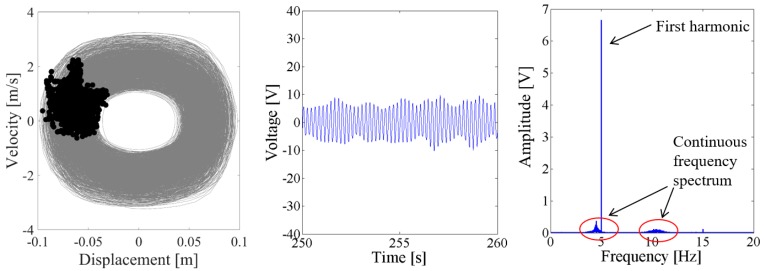
Dynamic responses of the coupled harvester-1 for the excitation level of 1.5 g.

**Figure 21:**
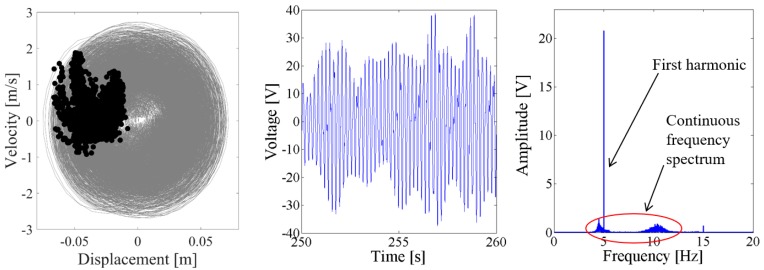
Dynamic responses of the coupled harvester-2 for the excitation level of 1.5 g.

Above results verify the energy harvesting enhancement of the presented NPEHS with two or three energy harvesters. In order to reveal its dynamic mechanism, nonlinear dynamic analysis is provided below. Figure 13 shows the bifurcation diagram of response voltages of the NPEHS-1 under zero initial conditions versus the excitation level. The excitation level ranging from 0 to 1.8 g is the control parameter to numerically simulate the stable response voltage of the NPEHS-1 with the excitation frequency of 5 Hz. It is found from Figure 13 that the NPEHS-1 may undergo periodic and chaotic responses along with the increase of the excitation level, which demonstrates its strong nonlinearity [66,67,71,72,73]. In purpose of checking these nonlinear dynamic response characteristics, the phase plane portrait of the response displacement and the response velocity, and its Poincare map, the time-domain output voltage and its spectrogram via fast Fourier transformation (the excitation frequency is 5 Hz) are shown in Figure 14, Figure 15, Figure 16, Figure 17, Figure 18, Figure 19, Figure 20 and Figure 21. In addition, the Poincare map is drawn by black dots.

When the excitation level is 0.1 g, Figure 14 and Figure 15 show that the output voltage of the coupled harvester-2 is larger than the coupled harvester-1, and the response voltage of the former is singly periodic. However, the Poincare map in the phase trajectory of the coupled harvester-1 consists of a series of discrete dots, as shown in the first plot of Figure 14. Its time-domain output voltage is the sine curve with fluctuating. The corresponding spectrum in Figure 14 shows that the fundamental harmonic is the uppermost component, while there is a non-ignorable 2.67 Hz fractional frequency component which modulates the fundamental harmonic.

As the excitation level increased to 0.6 g, the dynamic response of the NPEHS-1 is quite different with that when the excitation level is 0.1 g. In Figure 16, there are a lot of sub-harmonics and super-harmonics in the response voltage of the coupled harvester-1, as shown in the spectrum. Meanwhile, there are more different discrete dots in the Poincare map of the phase trajectory for the coupled harvester-2, as shown in Figure 17. In this case, the response of the coupled harvester-2 can be considered as chaotic response, which is confirmed by its spectrum.

As the excitation level is further increased to 0.9 g, the spectrum in both Figure 18 and Figure 19 show that there are the fundamental harmonic and the third harmonic in the response voltage of the two coupled energy harvesters. The Poincare map of the phase trajectory of the coupled harvester-1 looks like a fixed dot. This means that the response of the coupled harvester-1 is periodic, and there are no obvious sub-harmonics in the response voltage. However, there are continuous frequency components in the spectrum of the output voltage of the coupled harvester-2, and the fundamental harmonic is still the major component. The Poincare map of the phase trajectory of the coupled harvester-2 consists of a series of close dots, as the results shown in Figure 19.

When the excitation level is increased to 1.5 g, the response voltage is modulated by an infinite number of sub-harmonics and super-harmonics, because there are continuous frequency components in the spectrum of Figure 20 and Figure 21. The corresponding Poincare map of phase trajectory consists of a lot of discrete dots. Therefore, the responses of the NPEHS-1 at 1.5 g excitation are chaotic.

Above analysis demonstrates that the nonlinear dynamic response characteristics of the NPEHS change along with the excitation conditions, and its response may be periodic or chaotic depending on the excitation conditions and initial conditions. Meanwhile, the fundamental harmonic is an important component in the response voltage and the response displacement, and there are sub-harmonics and super-harmonics under some excitation conditions. These features can be used to enhance energy harvesting and sensing in the further study.

## 5. Conclusions

As an alternative nonlinear energy harvesting technique, a novel nonlinear piezoelectric energy harvesting system without magnets is presented in the purpose of enhancing vibration energy harvesting performance. A theoretical model is derived based on Euler-Bernoulli beam theory, piezoelectric theory, Kirchhoff’s law and the relevant geometrical relationship to predict the energy harvesting performance. Based on the appropriate design parameters, the output power area of the presented nonlinear system with two and three energy harvesters are 268.8% and 339.8% of their corresponding linear counterparts, respectively. This verifies that the presented nonlinear system can improve the energy harvesting efficiency under some specific conditions. In addition, nonlinear dynamic response characteristics are investigated by using the bifurcation diagram, the Poincare map of the phase trajectory, and the spectrum of the output voltage via fast Fourier transforms. The fundamental harmonic is found to be one main component of the response voltage, and sub-harmonics and super-harmonics are also found under some excitation conditions. This study focuses on the design, modeling and dynamic analysis of a novel nonlinear energy harvesting system for enhanced vibration energy harvesting. In the next step, further studies may focus on optimizing the number of energy harvesters, performing experiments and presenting the interface circuit for maximizing the energy harvesting performance.

## Figures and Tables

**Table 1:** Geometrical parameters (mm).

	*L*	*L_p_*	*L_c_*	*b*	*h_s_*	*h_p_*
Harvester-1	80	20	5	15	0.10	0.5
Harvester-2	80	20	5	15	0.18	0.5
Harvester-3	80	20	5	15	0.23	0.5

**Table 2:** Material property parameters.

**Substrate**		
**Parameter**	**Symbol**	**Value**
Young’s modulus	$E_{s}$	125 GPa
Density	$\rho_{s}$	8250 kg/m^3^
**Piezoelectric layers**		
Young’s modulus	$E_{p}$	63 GPa
Density	$\rho_{p}$	7700 kg/m^3^
Coupling coefficient	$d_{31}$	−285 × 10^−12^ C/N
Permittivity constant	$\varepsilon_{33}^{S}$	3200 $\varepsilon_{0}$
Permittivity of free space	$\varepsilon_{0}$	8.854 × 10^−12^ F/m

## Appendix A

Piezoelectric equations and boundary conditions:

The piezoelectric constitutive equations are used to describe the electromechanical behavior, defined as [3,74,75]:

(A1) $$T=c_{11}^{E}S-e_{31}E$$

(A2) $$D=e_{31}S+\varepsilon_{33}^{S}E$$

where *T* and *S* are the mechanical stress and the mechanical strain, respectively; *E* and *D* are the electric intensity and the electric displacement, respectively; $e_{31}$ is the electromechanical coupling coefficient; $c_{11}^{E}$ is the elasticity modulus measured in the zero electric field; $c_{33}^{S}$ presents s the piezoelectric material permittivity constant at zero strain condition.

At the clamped end, the displacement and the angle of rotation should be zero, which results in the following two equations for the boundary conditions:

(A3) $${\left(\phi (0)\right)}_{1}=0$$

(A4) $${\left(\phi (0)\right)}_{1}^{\prime }=0$$

Since the linear piezoelectric energy harvester is assumed to meet Euler-Bernoulli assumptions, the continuous condition of the displacement, the angle of rotation, the bending moment and the shear force are defined as:

(A5) $${\left(\phi (L_{p})\right)}_{1}={\left(\phi (L_{p})\right)}_{2}$$

(A6) $${\left(\phi (L_{p})\right)}_{1}^{\prime }={\left(\phi (L_{p})\right)}_{2}^{\prime }$$

(A7) $$EI_{1}{\left(\phi (L_{p})\right)}_{1}^{\prime \prime }=EI_{2}{\left(\phi (L_{p})\right)}_{2}^{\prime \prime }$$

(A8) $$EI_{1}{\left(\phi (L_{p})\right)}_{1}^{\prime \prime \prime }=EI_{2}{\left(\phi (L_{p})\right)}_{2}^{\prime \prime \prime }$$

At the free end, the tip mass block should be fully considered [35,66], and the boundary conditions are expressed as:

(A9) $$EI_{2}{\left(\phi (L)\right)}_{2}^{\prime \prime }-\omega^{2}M_{tip}L_{c}{\left(\phi (L)\right)}_{2}-\omega^{2}(I_{t}+M_{tip}L_{c}^{2}){\left(\phi (L)\right)}_{2}^{\prime }=0$$

(A10) $$EI_{2}{\left(\phi (L)\right)}_{2}^{\prime \prime \prime }+\omega^{2}M_{tip}{\left(\phi (L)\right)}_{2}+\omega^{2}M_{tip}L_{c}{\left(\phi (L)\right)}_{2}^{\prime }=0$$

where $I_{t}$ is the rotary inertia of the tip mass block.
